# Prognostic Significance of Tumor Size of Small Lung Adenocarcinomas Evaluated with Mediastinal Window Settings on Computed Tomography

**DOI:** 10.1371/journal.pone.0110305

**Published:** 2014-11-03

**Authors:** Yukinori Sakao, Hiroaki Kuroda, Mingyon Mun, Hirofumi Uehara, Noriko Motoi, Yuichi Ishikawa, Ken Nakagawa, Sakae Okumura

**Affiliations:** 1 Department of Thoracic Surgical Oncology, Cancer Institute Hospital, Japanese Foundation for Cancer Research, Tokyo, Japan; 2 Department of Thoracic Surgery, Aichi Cancer Center Hospital, Nagoya, Japan; 3 Department of Pathology, Cancer Institute Hospital, Japanese Foundation for Cancer Research, Tokyo, Japan; Memorial Sloan-Kettering Cancer Center, United States of America

## Abstract

**Background:**

We aimed to clarify that the size of the lung adenocarcinoma evaluated using mediastinal window on computed tomography is an important and useful modality for predicting invasiveness, lymph node metastasis and prognosis in small adenocarcinoma.

**Methods:**

We evaluated 176 patients with small lung adenocarcinomas (diameter, 1–3 cm) who underwent standard surgical resection. Tumours were examined using computed tomography with thin section conditions (1.25 mm thick on high-resolution computed tomography) with tumour dimensions evaluated under two settings: lung window and mediastinal window. We also determined the patient age, gender, preoperative nodal status, tumour size, tumour disappearance ratio, preoperative serum carcinoembryonic antigen levels and pathological status (lymphatic vessel, vascular vessel or pleural invasion). Recurrence-free survival was used for prognosis.

**Results:**

Lung window, mediastinal window, tumour disappearance ratio and preoperative nodal status were significant predictive factors for recurrence-free survival in univariate analyses. Areas under the receiver operator curves for recurrence were 0.76, 0.73 and 0.65 for mediastinal window, tumour disappearance ratio and lung window, respectively. Lung window, mediastinal window, tumour disappearance ratio, preoperative serum carcinoembryonic antigen levels and preoperative nodal status were significant predictive factors for lymph node metastasis in univariate analyses; areas under the receiver operator curves were 0.61, 0.76, 0.72 and 0.66, for lung window, mediastinal window, tumour disappearance ratio and preoperative serum carcinoembryonic antigen levels, respectively. Lung window, mediastinal window, tumour disappearance ratio, preoperative serum carcinoembryonic antigen levels and preoperative nodal status were significant factors for lymphatic vessel, vascular vessel or pleural invasion in univariate analyses; areas under the receiver operator curves were 0.60, 0.81, 0.81 and 0.65 for lung window, mediastinal window, tumour disappearance ratio and preoperative serum carcinoembryonic antigen levels, respectively.

**Conclusions:**

According to the univariate analyses including a logistic regression and ROCs performed for variables with p-values of <0.05 on univariate analyses, our results suggest that measuring tumour size using mediastinal window on high-resolution computed tomography is a simple and useful preoperative prognosis modality in small adenocarcinoma.

## Introduction

We previously reported that the size of lung adenocarcinoma, evaluated using mediastinal window (MD) settings on computed tomography (CT), is a more important predictive prognosis factor than the total tumour size, evaluated using lung window (LD) settings [1] Various studies have documented the correlation between CT findings and the pathological features of lung adenocarcinoma [2]–[4]. The ground glass opacity (GGO) component is typically recognized as a bronchioloalveolar carcinoma (BAC) component on microscopic examination, and the BAC is now categorized as an adenocarcinoma in situ that does not affect tumour aggressiveness [5], [6]. In contrast, the solid component recognized as invasive lesion being so called scar, which excludes the BAC component in lepidic predominant adenocarcinoma, can be easily defined using MD settings on CT [1], [2], [5]. Moreover, the solid tumour recognized as a non-lepidic predominant adenocarcinoma, such as acinar, papillary, solid predominant or micropapillary predominant adenocarcinomas, is recognized as invasive adenocarcinoma and shows much more aggressiveness than that by lepidic predominant adenocarcinoma [2], [5], [7]. Therefore, we have emphasized the importance of determining the size of the solid tumour component in adenocarcinoma using MD settings when evaluating tumour aggressiveness [1], [2], [5].

This investigation aimed to clarify the importance of the tumour size evaluated by MD settings as a preoperative prognostic predictive factor for anatomical pulmonary resection in patients with small adenocarcinomas (1–3 cm). Furthermore, we would clarify that the preoperative evaluation of tumour diameter by CT with MD settings would enable the prediction of prognosis, lymph node metastasis and tumour invasiveness for patients with clinically early-stage tumours.

## Materials and Methods

This was a retrospective study conducted between October 2003 and December 2008 in patients with small lung adenocarcinomas (diameters of ≤3 cm) that underwent standard surgical resections (lobectomy with hilar and mediastinal lymph node dissection) at the Cancer Institute Hospital.

Tumour dimension was evaluated under two different CT imaging conditions: LD [level  = −500 Hounsfield unit (HU), width  = 1500 HU] and MD (level  = 60, width  = 350 HU). The CT (multi-detector CT, Toshiba, Japan) images were evaluated for the maximum tumour dimension.

Tumour disappearance ratio (TDR) was defined as 1− MD/LD.

“For all patients, preoperative staging was assessed using chest CT, CT or ultrasonography for abdominal metastasis, brain CT or magnetic resonance imaging for the brain metastasis and bone scanning for bone metastasis.”

Clinical mediastinal and hilar lymph node status was deemed positive if the chest CT findings revealed a lymph node short axis of >1.0 cm. The status of mediastinal, hilar or interlobar nodes was assessed according to the classification for lung cancer in the TNM Classification of Malignant Tumours, Seventh Edition [8]. The CT findings were reviewed by two independent radiologists

We excluded tumours comprising 100% GGO from this study because most of them were believed, on microscopic examination, to be non-invasive or precancerous lesions. The GGO component was defined as hazy and amorphous with increased lung attenuation, but without obscuration of the underlying vascular markings and bronchial walls. In addition, we excluded a subgroup with BAC or mucinous BAC on microscopic examination that were defined as adenocarcinoma in situ [6]. We also excluded tumours measuring <1 cm because they were few in number and did not undergo standard resections. To select this cohort, the tumour size was used measured by two independent radiologists with CT in LD. Of total 246 patients with small lung adenocarcinomas (diameters of ≤3 cm) who underwent standard surgical resections, 36 were excluded due to lack of thin slice data in CT, 24 were excluded due to adenocarcinoma in situ and 10 were excluded due to size smaller than 1 cm. Therefore, 176 patients were examined in this study.

Patient records were examined for age, gender, preoperative nodal status and tumour size, as evaluated using both MD and LD. Preoperative serum carcinoembryonic antigen (CEA) levels, TDR and pathological status were evaluated using elastic stain, and included lymphatic vessel (ly), vascular vessel (v) and pleural (pl) invasion.

Because individual patients were not identified, our institutional review board (Review Board in Cancer Institute Hospital, Japanese Foundation for Cancer Research) approved this study without the requirement to obtain patient consent. The patient records/information was anonymized and de-identified prior to analysis.

### Statistical Analyses

Disease-free survival was assessed. Survival duration was defined as the interval between surgery and either tumour relapse or the most recent follow-up. The Kaplan–Meier method was used to calculate the recurrence-free survival rates. Univariate analyses included a log-rank test, chi-square test and logistic regression. Receiver operating characteristic analyses (ROC) were performed for variables with *P*-values of <0.05 on univariate analysis using the logistic regression test or the Cox proportional hazards model. All analyses were performed using the JMP 10 software (SAS Institute Incorporated, Cary, North Carolina) and results with *P*-values of <0.05 were considered statistically significant.

## Results

In total, 176 patients were enrolled. This subgroup that excluded BAC and small tumours (<1 cm) comprised 99 females and 77 males, with ages ranging from 34 to 78 (median  = 61) years. The follow-up periods ranged from 24–84 (median  = 49) months.

### Preoperative prognostic factors for disease-free survival

**Figure 1:**
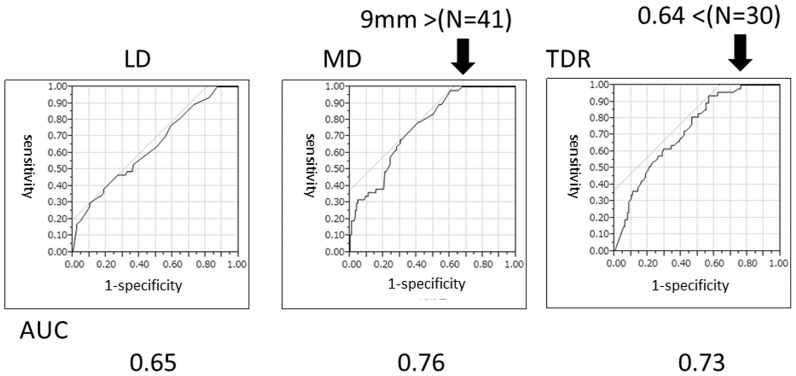
Receiver operating characteristic analyses for recurrence. Tumour dimension was evaluated using lung window (LD) and mediastinal window (MD) settings. TDR: tumour disappearance ratio (TDR  = 1− MD/LD). Allow indicated a value at 100% sensitivity.

**Figure 2:**
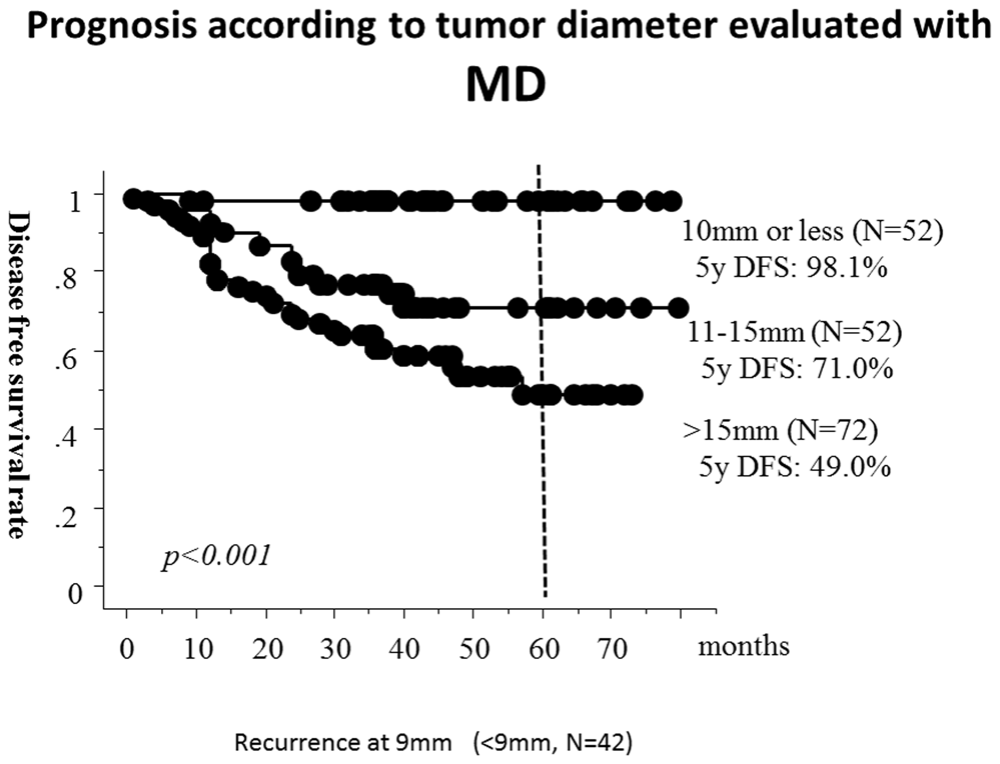
The 5-year disease-free survival curve according to tumour dimension using mediastinal window (MD) settings. Tumour dimension was evaluated using mediastinal window (MD) settings. One case with MD 9 mm showed recurrence and the case is the smallest in MD among all.

As shown in Table 1, LD findings, MD findings, TDR and nodal status (cN) were significant prognostic factors for disease-free survival on univariate analyses. The AUCs for recurrence were 0.76, 0.73 and 0.65, for MD, TDR and LD, respectively (Figure 1).The 5-year disease-free survival rates according to MD were 98.1% for ≤10 mm (N = 52), 71.0% for 11–≤15 mm (N = 52) and 49.0% for>15 mm (N = 72). (*P*<0.001) (Figure 2).

**Table 1:** Preoperative prognostic factors for disease-free survival with small adenocarcinomas (≤3 cm).

Variables	Odds ratio	95% CI	*P* value
Gender (female)	0.61	0.34–1.07	.08
Age	0.99	0.97–1.03	.74
LD	1.08	1.02–1.14	.007
MD	1.13	1.08–1.18	<.001
TDR	38.6	7.35–	<.001
CEA	0.33	0.99–1.03	.33
High (>5 ng/ml)/normal)	1.89	0.96–3.72	.066
cN (cN0/cN1-2)	0.23	0.11–0.50	<.001

Logistic regression test (Univariate analyses).

LD: diameter using lung window setting, MD: diameter using mediastinal window setting, RFS: recurrence-free survival, TDR: tumour disappearance ratio (TDR  = 1− MD/LD), CEA: carcinoembryonic antigen, cN: preoperative nodal status, CI: confidence interval.

### Preoperative Factors Associated with Lymph Node Metastasis

**Figure 3:**
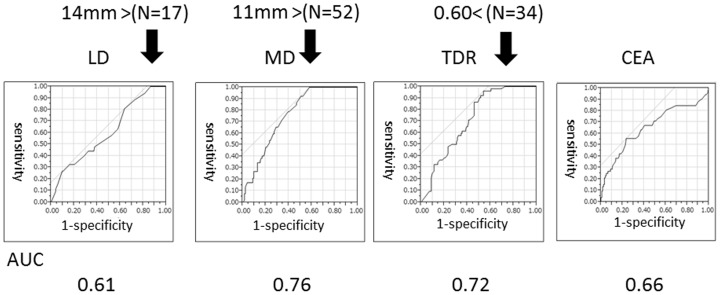
Receiver operating characteristic analyses for lymph node metastasis. Tumour dimension was evaluated using lung window (LD) and mediastinal window (MD) settings. TDR: tumour disappearance ratio (TDR  = 1− MD/LD), CEA: carcinoembryonic antigen. Allow indicated a value at 100% sensitivity.

**Figure 4:**
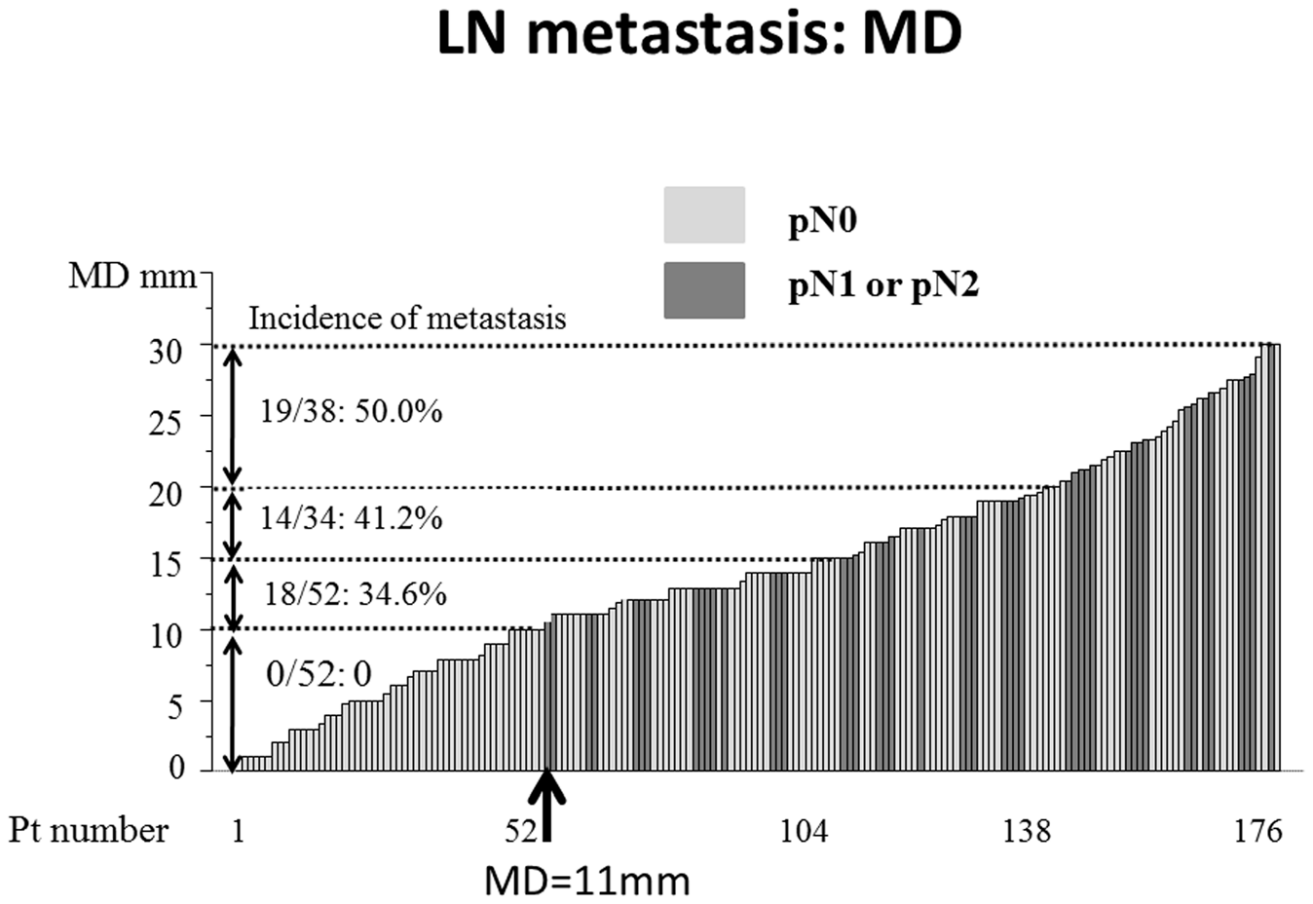
Incidence of lymph node metastasis according to tumour dimension using mediastinal window (MD) settings. Tumour dimension was evaluated using mediastinal window (MD) settings. A black bar showed a patient with lymph node metastasis and a gray bar showed a patient without lymph node metastasis.

As shown in Table 2, LD findings, MD findings, TDR, CEA levels and cN were significant factors associated with lymph node metastasis on univariate analyses. The area under the curves (AUCs) for lymph node metastases were 0.61, 0.76, 0.72 and 0.66 for LD, MD, TDR and CEA, respectively (Figure 3). The incidence of lymph node metastases according to MD were 0% for <10 mm (N = 52), 34.6% for>10 mm–15 mm(N = 52), 41.2% for>15 mm–20 mm (N = 34) and 50.0% for>20 mm (N = 38) (Figure 4).

**Table 2:** Preoperative factors associated with lymph node metastasis in small adenocarcinoma (≤3 cm).

Variables	Odds ratio	95% CI	*P* value
Gender (female)	0.66	0.34–1.28	.22
Age	1.03	0.99–1.03	.86
LD	1.07	1.01–1.15	.03
MD	1.15	1.09–1.21	<.001
TDR	66.7	10.0–	<.001
CEA	1.11	1.01–1.12	.01
High (>5 ng/ml)/normal)	3.0	1.31–6.87	.009
cN (cN0/cN1-2)	30.3	3.76–250	<.001

Logistic regression test (Univariate analyses).

LD: diameter by lung window setting, MD: diameter by mediastinal window setting, TDR: tumour disappearance ratio (TDR  = 1− MD/LD), CEA: carcinoembryonic antigen, cN: preoperative nodal status, CI: confidence interval, LN: lymph node.

### Preoperative factors associated with lymphatic vessel, vascular vessel or pleural invasion in small adenocarcinomas

**Figure 5:**
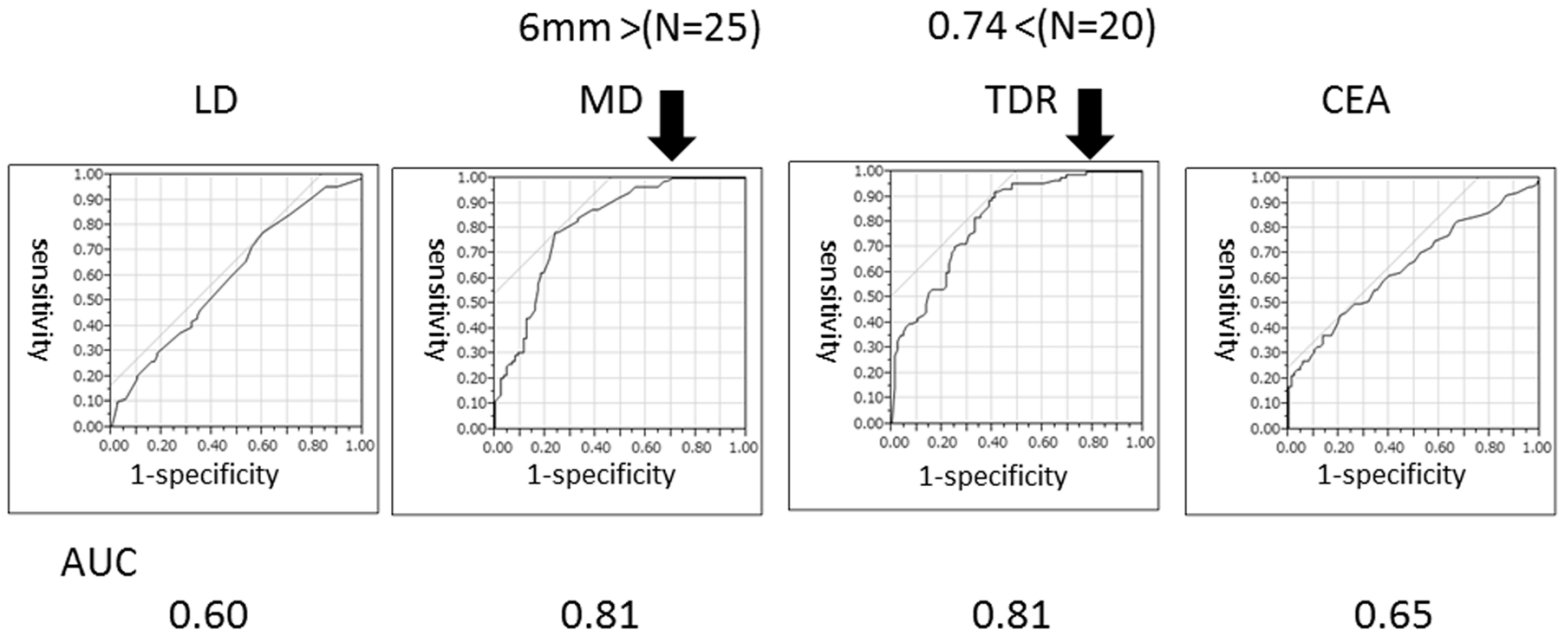
Receiver operating characteristic analyses for pleural, lymphatic and vascular invasion. Tumour dimension was evaluated using lung window (LD) and mediastinal window (MD) settings. TDR: tumour disappearance ratio (TDR  = 1− MD/LD), CEA: carcinoembryonic antigen. Allow indicated a value at 100% sensitivity.

**Figure 6:**
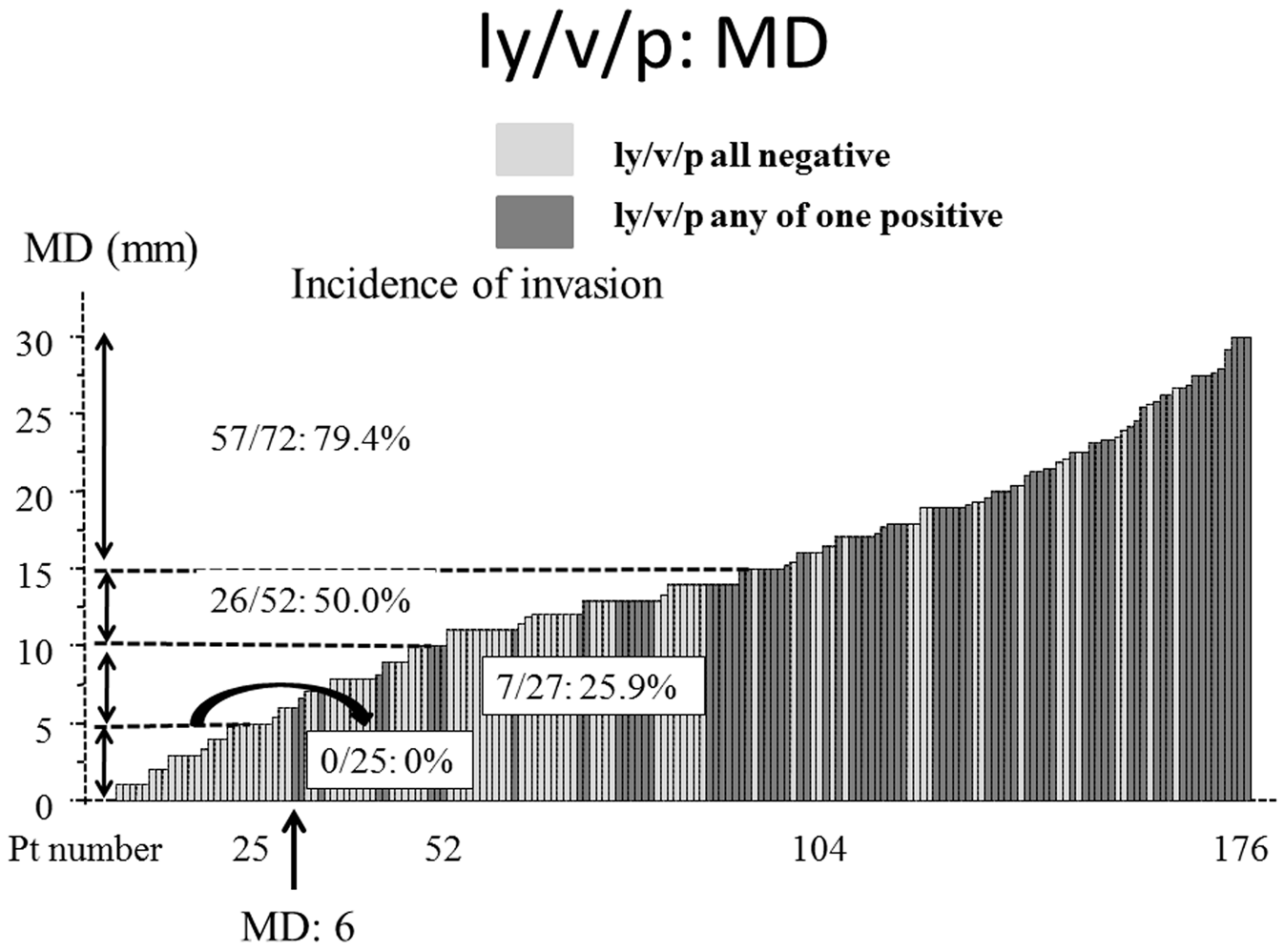
Incidence of lymphatic vessel, vascular vessel or pleural invasion in small adenocarcinomas according to tumour dimension using mediastinal window (MD) settings. A black bar showed a patient with invasion to any of lymphatic vessel, vascular vessel or pleura, and a gray bar showed a patient without any of them.

As shown in Table 3, LD findings, MD findings, TDR, CEA levels and cN were significant factors for lymphatic vessel, vascular vessel or pleural (ly/v/pl) invasion on univariate analyses. The AUCs for ly/v/pl invasion were 0.60, 0.81, 0.81 and 0.65 for LD, MD, TDR and CEA, respectively (Figure 5). The incidence of ly/v/pl invasion according to MD were 0% for ≤5 mm (N = 25), 25.9% for>5 mm–10 mm (N = 27), 41.2% for ≥10 mm–15 mm (N = 52) and 79.4% for>15 mm(N = 72) (Figure 6).

**Table 3:** Preoperative factors associated with pleural, lymphatic and vascular invasion in small adenocarcinomas (≤3 cm).

Variables	Odds ratio	95% CI	*P* value
Gender (female)	0.66	0.36–1.20	.17
Age	1.00	0.97–1.03	.87
LD	1.06	1.00–1.12	.03
MD	1.21	1.14–1.30	<.001
TDR	6.76	3.50–13.2	<.001
CEA	1.23	1.07–1.40	.002
High (>5 ng/ml)/normal)	5.9	2.12–16.3	<.001
cN (cN0/cN1-2)	11.1	1.40–90.9	.03

Logistic regression test (Univariate analyses).

LD: diameter by lung window setting, MD: diameter by mediastinal window setting, TDR: tumour disappearance ratio (TDR  = 1− MD/LD), CEA: carcinoembryonic antigen, cN: preoperative nodal status, CI: confidence interval, ly: lymphatic vessels, v: vascular vessels, pl: pleura.

## Discussion

Tumour diameter is a major prognostic factor for lung cancer. The most common method for determining tumour size before surgery is by CT using lung window settings [8]. Recently, attempts were made to classify small peripheral adenocarcinomas into subgroups according to the patterns of tumour growth, which are considered to be associated with the biological characteristics of tumours derived from clinicopathological examination[4], [6], [9]–[11]. These subgroups comprise the following: AIS (adenocarcinoma in situ), minimally invasive adenocarcinoma (3-cm lepidic predominant tumour with an invasion of ≤5 mm), lepidic predominant, acinar predominant, papillary predominant, micropapillary predominant, solid predominant with mucin production and invasive adenocarcinoma variants.

It has been reported that tumour size was not associated with either indicators of proliferation or tumour invasiveness [1], [2], [5], [9], [11]. In fact, histological subgrouping based on growth patterns provides a clear indication of the biological characteristics of peripheral small lung adenocarcinoma than simple lesion size [7], [9]. That is, papirally, acinar, micropapillery or solid predominant adenocarcinoma have more aggressive character than lepidic predominant adenocarcinoma when tumoue size is limited to 3cm or smaller [7]–[11].

Furthermore, it has been reported that the larger or more desmoplastic fibrous scars is asosiated with more aggressive tumour invasion and poorer prognoses [7], [9]–[11]. In addition, we previously reported that tumour size, excluding the BAC component (lepidic growth), was an important indicator of tumour invasiveness and that tumour diameter evaluated using MD was associated with the tumour growth pattern (i.e. non-lepidic growth component) and scars [1], [2], [5]. The solid lesion evaluated with MD in CT is the tumor lesion without lepidic growth adenocarcinoma. As an easy and simple extracting method of the solid area representing an invasive lesion (scar) of the adenocarcinoma or non-lepidic predominant adenocarcinoma (invasive adenocarcinoma), we examined the usefulness of tumour size as evaluated using MD. In other words, using LD to evaluate the total tumour diameter cannot directly detect the solid lesion associated with tumour aggressiveness, but can detect the associated BAC (lepidic growth) component, which is not associated with tumour aggressiveness [1], [2], [5], [14].

In the present study, we confirmed that tumour dimensions determined using MD settings provided additional useful prognostic data that could not be evaluated using LD settings [1], [2], [5]. Furthermore, tumour size was a significantly better prognostic factor when evaluated using MD instead of LD. The MD were an important predictive factor for prognosis as well as for lymph node involvement and tumour invasiveness in small lung adenocarcinoma (1–3 cm). A new concept −minimally invasive adenocarcinoma (MIA)− has been proposed. This is a small, solitary adenocarcinoma (≤3 cm) with a predominantly lepidic pattern and invasion of ≤5 mm in the greatest dimension in any one focus. Patients with an MIA have a nearly 100% disease-specific survival if it is completely resected. The invasive component to be measured in an MIA is defined as follows: (1) histological subtypes other than a lepidic pattern (i.e., acinar, papillary, micropapillary, and/or solid) or (2) tumor cells infiltrating into myofibroblastic stroma. MIA is excluded if the tumor (1) invades lymphatics, blood vessels, or pleura or (2) has tumor necrosis [6]. Accordin to the present study, when the MD findings were equal or smaller than 5 mm, no patient showed vessel invasion, plural invasion, or tumor relapse. Therefore, MD may be a promising criteria to be used for CT classification as the newly proposed minimally invasive adenocarcinoma.

Tumour markers are used clinically to assist in the diagnosis of patients with non-small-cell lung carcinoma and to monitor progression, recurrence and/or efficiency of treatment. Among these markers, CEA is considered to be the most prevalent marker for the diagnosis and monitoring of lung adenocarcinoma. Serum CEA levels are an important prognostic factor for early-stage adenocarcinoma, such as clinical stage IA tumours [12]–[14]. In the present study, univariate analysis revealed that CEA levels were correlated with lymph node metastasis and v or pl invasion, with a tendency for predicting postoperative prognosis. However, CEA levels were not a significant prognostic factor for lymph node metastasis following multivariate analyses with MD findings. This may be explained by the high correlation between CEA levels and MD findings. When CEA levels were compared among the tumour size groups, they gradually and significantly increased with tumour stage progression (*P*<0.01). When the mediastinal size was>20 mm, one-fourth of the patients in this cohort showed serum CEA levels beyond cut off value at 5 ng/ml.

TDR is an established and important prognostic factor. Suzuki et al. reported that radiological non-invasive (neither ly nor v) peripheral lung adenocarcinoma could be defined as an adenocarcinoma of ≤2.0 cm with ≤0.25 consolidation [15]. The results of our study were similar to those of a previous study on TDR [16.17].

The solid type adenocarcinoma on CT was highly associated with non-lepidic predominant adenocarcinoma, such as acinar predominant, papillary predominant and solid predominant with mucin production [2], [3], [5]. In invasive adenocarcinoma, lepidic predominant adenocarcinomas have a much better prognosis than non-lepidic predominant adenocarcinomas [1], [2], [5], [9]–[11]. It was therefore suggested that the size of a non-lepidic tumour component evaluated using MD findings on CT was a useful indicator of solid adenocarcinoma aggressiveness.

In this study, the patient population was limited to those with tumour diameters of 1–3 cm. Patients with tumour diameters of <1 cm were excluded because of the small number of cases and the major bias to perform wedge resection. Another problem was the LD and MD imaging conditions for CT. According to 7^th^ general rule for clinical and pathological record of lung cancer, the recommended LD are a window level of −500 to −700 and a window width of 1000–2000 HU, whereas we used −500 HU and 1500 HU, respectively. The recommended MD are a window level of 30–60 H and a window width of 350–600 HU, while we used 60 HU and 350 HU, respectively.[8]

The differences in these imaging conditions for CT may have affected the MD, LD and TDR results. Furthermore, the solid component size defined as a lesion without GGO evaluated by LD is a promising method of the solid area representing an invasive lesion (scar) of the adenocarcinoma,

Therefore, further investigations on the optimal CT imaging conditions for assessing the aggressiveness of small adenocarcinomas should be performed in multiple centres with larger sample sizes. The size of the solid lesion (excluding the GGO component) evaluated using LD is also a useful CT-based prognostic factor. Further examination is necessary to clarify the relative utility of both tumour size measured using MD and extracted solid size measured by LD.

In conclusion, our results suggest that measuring tumour size with MD on high-resolution CT is a simple and useful preoperative modality for predicting invasiveness, lymph node metastasis and prognosis. An increased tumour size with MD correlated with pathological malignant potential reflecting tumour aggressiveness and degree of invasion.
